# Investigating Health Equity and Healthcare Needs among Immigrant Women Using the Association Rule Mining Method

**DOI:** 10.3390/healthcare9020195

**Published:** 2021-02-10

**Authors:** Ming-Hseng Tseng, Hui-Ching Wu

**Affiliations:** 1 Department of Medical Informatics, Chung Shan Medical University, Taichung 40201, Taiwan; mht@csmu.edu.tw; 2 Department of Medical Sociology and Social Work, Chung Shan Medical University, Taichung 40201, Taiwan; 3 Social Service Section, Chung Shan Medical University Hospital, Taichung 40201, Taiwan

**Keywords:** immigrant women, health inequality, healthcare needs, association rule mining algorithm, health policies

## Abstract

Equitable access to healthcare services is a major concern among immigrant women. Thus, this study investigated the relationship between socioeconomic characteristics and healthcare needs among immigrant women in Taiwan. The secondary data was obtained from “Survey of Foreign and Chinese Spouses’ Living Requirements, 2008”, which was administered to 5848 immigrant women by the Ministry of the Interior, Taiwan. Additionally, descriptive statistics and significance tests were used to analyze the data, after which the association rule mining algorithm was applied to determine the relationship between socioeconomic characteristics and healthcare needs. According to the findings, the top three healthcare needs were providing medical allowances (52.53%), child health checkups (16.74%), and parental knowledge and pre- and post-natal guidance (8.31%). Based on the association analysis, the main barrier to the women’s healthcare needs was “financial pressure”. This study also found that nationality, socioeconomic status, and duration of residence were associated with such needs, while health inequality among aged immigrant women was due to economic and physical factors. Finally, the association analysis found that the women’s healthcare problems included economic, socio-cultural, and gender weakness, while “economic inequality” and “women’s health” were interrelated.

## 1. Introduction

Equitable access to healthcare services is an important policy issue, especially for achieving positive health outcomes. However, unequal accessibility to such services among immigrant populations is a frequent occurrence. Meanwhile, gender is a multi-dimensional social structure that not only embodies social relations that encompass material, discursive, and effective relations but also operates on cognitive, interpersonal, and institutional levels. As immigrant women’s healthcare needs are important for promoting health equity, the present study investigates Taiwanese immigrants’ experiences in accessing healthcare, including the barriers that immigrant women face when accessing such services.

In general, the immigration process entails many changes in the lives of those who emigrate, including establishing one’s identity in a new country. Some immigrants experience poor mental or emotional health because of the impact of social integration, socioeconomic status, language barriers, diverse cultural perspectives toward healthcare, information barriers, and financial difficulties [1,2,3,4,5,6]. As for other aspects, the relationship between gender and health is a dynamic process. In this regard, immigrant women’s health is especially determined by personal (physical and mental), social, economic, and environmental factors (e.g., education, working conditions, social status, and social support networks).

Based on our research findings, social status and financial stress deeply affect immigrant women’s health affordability. Thus, this study:

1. Suggests a relationship between socioeconomic characteristics and healthcare needs among immigrant women in Taiwan, and

2. Utilizes association rule models along with different socioeconomic status conditions to investigate immigrant women’s healthcare needs.

### 1.1. Immigrant Women’s Health Equity

Immigration and adaptation to the host culture should be considered as serious life event changes. In fact, numerous studies have identified stresses that have potentially negative consequences on immigrants’ health, including finding employment and maintaining an income source, establishing a new home, feelings of loss of social status, and social isolation. These stresses are also significantly affected by language barriers [1,7,8,9,10,11]. Some cross-cultural psychological studies have found important connections between cultural contexts and individual behavioral development. The long-term psychological consequences of this acculturation process are highly variable, depending on social and personal variables that reside in the society of origin, the society of settlement, and the phenomena that exist prior to and during the course of acculturation [11]. Additionally, the majority of immigrant groups that arrive in industrialized countries are economically disadvantaged with respect to the native-born population, thus experiencing lower levels of income, barriers to healthcare access and utilization, and higher levels of discrimination, all of which contribute to adverse health outcomes. Moreover, some researchers have reported that the health advantage of immigrants declines after migration, both for first-generation migrants [12,13] and subsequent generations [14,15]. In comparison with examining immigrants’ medical care needs, investigating their healthcare needs may help in understanding the social determinants of health and promote immigrants’ health equity [16].

### 1.2. Healthy Immigrant Effect and Health Stratification

Immigrants from industrialized countries generally present better health outcomes (e.g., mental health, mortality, and pregnancy outcomes) than the native-born population, i.e., the concept of the “healthy migrant effect” [17]. It has also been suggested that this effect may be conceptualized as a “recent immigrant” phenomenon. Previous research has shown that the regression of this effect is associated with the number of years living in industrialized countries [13,18,19,20] and that immigrants’ duration of residence may modify the disparities in pregnancy outcomes [12]. Selective immigration policies can also partly explain the phenomenon of “recent immigrant advantage.” However, this advantage is not retained by immigrants over the long term.

There are some conditions regarding immigrants’ levels of exposure to the receiving society that must be considered. First, newly married female immigrants tend to be progressively assimilated into the receiving society. For example, in most countries, health examinations concerning pre-term birth and morbidity during pregnancy are supported by government policies. During this period, immigrant mothers are healthy because of the benefits of health examinations, but their native health behaviors are lost because of acculturation. Second, it is likely that changes in immigrants’ health outcomes will be associated with the number of years they have lived in the receiving society. However, such trends will not necessarily be similar to the native-born population. In fact, they may even exceed those of the population [19]. In general, an ethnic minority is at a disadvantage in terms of health not only because of immigration but also because of duration of residence. This inconsistency has been referred to as the “acculturation paradox” [21]. Third, some researchers have reported that other health conditions, such as gestational diabetes and severe maternal morbidity, are more common among immigrant groups, thus suggesting that the existence of the healthy migrant effect may depend on other factors [22,23,24,25]. In this regard, the examination of multiple pregnancy outcomes and associated risk factors according to women’s exposure to the receiving society may provide a more comprehensive picture and help gain valuable insights into the immigrant paradox in reproductive health.

Based on the number of years they have lived in the new country and their financial ability, the immigrant paradox reveals that new immigrants simultaneously exhibit some of the most favorable outcomes (e.g., the lowest prevalence of pre-term delivery and morbidity during pregnancy) and the most unfavorable socioeconomic conditions (e.g., the highest prevalence of low income and low support). Some immigrants even appear to worsen their health status when compared with native residents. The loss of the healthy immigrant effect in the context of an economic crisis appears to be a possible explanation [26]. Moreover, low income can lead to poor lifestyles that eventually have negative impacts on health and well-being [1,27].

Previous research on the determinants of poverty among aged women has documented socioeconomic and demographic correlations among the poor and has examined the financial impact of adverse later-life events, such as widowhood, deterioration of health, and loss of employment. The financial resources available in old age depend mainly on previous long-term financial status through the majority of adult life [28,29]. The factors contributing to older women’s economic well-being from a life course perspective could be investigated based on midlife characteristics, such as workforce participation, income, and rural residence [30]. Women’s poverty is often a result of the gender gap and female dependence reflected in the gender division of labor, such as in employment and family relationships [31]. Therefore, a substantial gender gap in financial security during old age remains, making women more dependent than men on government policies. Social policy plays an important role in providing for women’s economic security [32].

## 2. Methods

### 2.1. Data Source

The secondary data in this study were obtained from the “Survey of Foreign and Chinese Spouses’ Living Requirements, 2008,” which was administered by the Ministry of the Interior, Taiwan [33]. The data distribution was performed by Academia Sinica, a survey research data archive.

The official survey focused on basic personal attributes, the status of family members, employment, healthcare needs, life adaptation, and living environments. Using the proportional systematic sampling method, the target population number was 407,487, of which 13,345 surveys were completed. After excluding 7497 missing values, the total number was 5848. The present study’s protocol was approved by the Institutional Review Board of Chung Shan Medical University Hospital (Permission No. CS13049, Waiver of Informed Consent).

### 2.2. Association Rule Mining

In general, large amounts of data are routinely gathered during day-to-day management in medicine and the delivery of health services. To mine useful information and knowledge from these databases, an important research area called “data mining” has been developed. There are many tasks associated with this area, such as classification, clustering, regression, and association rule mining [34].

Association rule mining [35,36] is one of the most popular data mining techniques. Its purpose is to extract a set of highly correlated features that are shared among a large number of records in a given database. For example, the rules found from a sales database can be useful for decision-making for marketing managers. The application of these association rules is also helpful in market basket analysis. For instance, it helps decision makers analyze customers’ purchase habits by discovering associations among various items.

Interestingly, Agrawal et al. [36] developed the Apriori algorithm for solving the association rule mining problem. In this regard, a rule is only defined between a set and a single item [30]. Moreover, every rule is composed of two different sets of items (or itemsets): X and Y, where X is the antecedent or left-hand-side (LHS) and Y is the consequent or right-hand-side (RHS). To determine whether the identified rules are meaningful, there are three quality measurements: support, confidence (reliability), and lift (correlation). The general idea is that if, for example, XY are frequent itemsets, we can determine if the association rule X→Y is meaningful by calculating support, confidence, and lift.

As for the measurements themselves, support (X→Y) is the fraction of the numbers in the database containing XY, which implies the frequency of occurring patterns. Meanwhile, confidence is an indication of how often the rule has been found to be true. Additionally, confidence refers to the ratio of support (X→Y)/support (X) and measures the strength of the implications. Moreover, lift (X→Y) is calculated as the ratio of confidence (X→Y)/support (Y) and measures the correlation between itemsets XY.

In this process, if a rule includes a lift of 1, it implies that the probability of occurrence of the antecedent and that of the consequent are independent of one another. In this case, no rule can be drawn involving these two events. However, if the lift is larger than 1, it implies the degree to which these two occurrences are dependent on one another. This also makes these rules potentially useful for predicting the consequent in future datasets. Meanwhile, if the lift is smaller than 1, it means that the presence of one item has a negative effect on the presence of the other item and vice versa.

In this study, the package “arules” [37,38] in R software [39] was the data mining method applied to explore the association rule mining of the relationship between immigrant women’s characteristics and healthcare needs. In this case, we extracted high-quality association rules using support (X→Y) > 0.05, confidence (X→Y) > 0.8, and lift (X→Y) > 1, after which the results of 26 association rules were found, respectively.

Finally, as it is difficult to show all the rules merely through descriptions, visualization can be effectively used to communicate both abstract and concrete ideas. This has been especially helpful in the fields of education, science, and engineering [40]. The most frequently used visualization techniques for analyzing association rules are scatter plots, matrix visualizations, graphs, mosaic plots, and parallel coordinate plots [41]. In the present study, we visualized the association rules using the scatter plots and graph-based visualizations implemented in the package “arules Viz” [37].

## 3. Results

In this study, SPSS software was used for the statistical analysis, while R open-source software was employed for the association analysis. The results are as follows.

### 3.1. Descriptive Statistics

Table 1 shows the healthcare needs of the 5848 participants after excluding the data designated as “other” or “missing value.” In the theme “healthcare needs,” the questionnaire items included “provide medical allowances,” “provide child health checkups,” “provide parental knowledge and pre- and post-natal guidance,” “provide knowledge of infectious diseases,” “assist medical communication,” “help joining the national health insurance program,” “provide reproductive health knowledge,” and “provide information of pre-natal medical allowances.” Overall, the top three items for immigrant women were “provide medical allowances” (52.53%), “provide child health checkups” (16.74%), and “provide parental knowledge, and pre- and post-natal guidance” (8.31%).

As presented in Table 2, 2238 (38.27%) women came from Southeast Asia, Hong Kong, Macao, and other countries, while 3610 (61.73%) came from mainland China. When asked about the item “the number of years lived in Taiwan,” their responses included “<4 years” (1334; 22.81%), “4–6 years” (1064; 18.19%), “6–8 years” (1291; 22.08%), and “>8 years” (2154: 36.83%). Regarding “marriage frequency,” 5040 (86.18%) were married once, while 783 (13.39%) were married twice or more. When asked about “the main source of spending money,” 3709 (63.42%) mentioned family members (e.g., husband, parents of the husband, and children), whereas only 1921 (32.85%) mentioned work income and savings. Regarding the educational level of their husbands, 793 (13.56%) mentioned that they were illiterate, self-learned, or had primary school education, 1622 (27.74%) had junior high school education, 2378 (40.66%) had senior high school education, and 990 (16.93%) had college, university, or graduate education.

Clearly, the statistics in Table 2 demonstrate that the healthcare needs (Y) of the immigrant women (with a confidence level of more than 95%) were significantly related with the 13 socioeconomic characteristics (X). Conversely, only “the main source of family living expenses” and “people around me are very friendly” were not significantly associated with their healthcare needs, respectively (with a confidence level of less than 95%). In other words, these two variables are independent of immigrants’ healthcare needs. Even though immigrant women had different sources of family living expenses and different feelings of community relationship, these two variables were not significantly associated with their healthcare needs statistically.

### 3.2. Association Rule Models

This study used the association rule mining algorithm to investigate the relationship between socioeconomic characteristics (X) and healthcare needs (Y) among immigrant women in Taiwan (see Table 3). In this case, “X” is the composite of the 45 attribute values of the immigrant women, including “nationality,” “education degree,” “type of identity documents,” “duration of residence in Taiwan,” “marriage frequency,” “the main source of pocket money,” “educational level of husband,” “marriage frequency of husband,” “does husband have a job?” “average income of husband,” “the number of births with Taiwanese husband,” “the main source of family living expenses,” “people around me are very friendly,” “I live in a safe place; disasters and accidents are rare,” and “overall, I am very happy in Taiwan.” Meanwhile, “Y” includes three main items: “providing medical allowances,” “providing child health checkups,” and “providing parental knowledge and pre- and post-natal guidance.”

**Figure 1:**
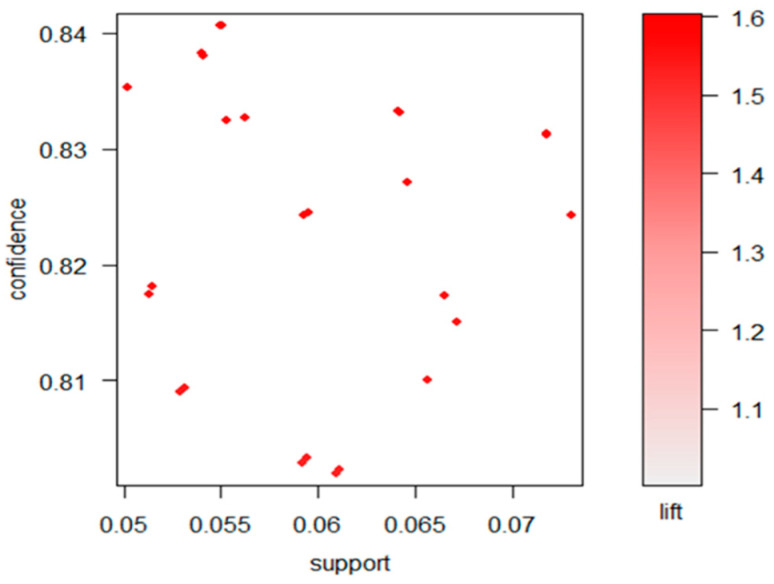
Scatter plot for the 26 rules.

Table 4 presents the results of the 26 association rule models with different socioeconomic status conditions (X) and healthcare needs (Y) for the immigrant women (A45: provide medical allowances). The range of the three quality measurements for the 26 association rule models were “0.050~0.073” for support, “0.802~0.841” for confidence, and “1.526–1.600” for lift. A scatter plot was also created to display a straight-forward visualization of the association rules using support and confidence on the axes. Additionally, a third measure (lift) was used as the color (red level) of the points. A color key is provided to the right of the plot. As shown in Figure 1, we can see that the rules with high lift had relatively low support, with the most interesting rules residing on the support/confidence border. Later, we will show how the interactive features of this plot can be used to explore these rules.

**Figure 2:**
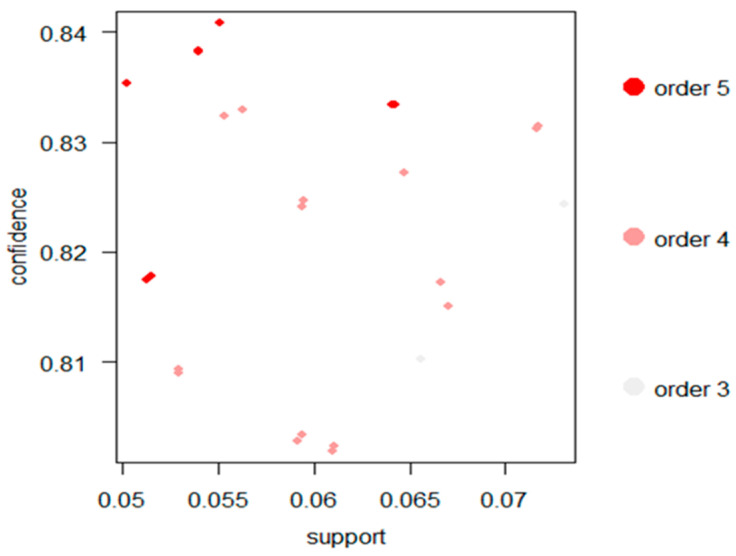
Two-key plot for the 26 rules.

Figure 2 presents the two-key plot for the 26 rules. In this plot, introduced by Unwin et al. [42], support and confidence are represented by the x and y axes, while the color of the points is used to indicate “order,” i.e., the number of items contained in the rule. As shown in this figure, order and support have a slightly stronger reverse relationship, which indicates that confidence and lift will be in accordance with order. Overall, the 26 rules present a nearly uniform distribution on the two-key plot.

## 4. Discussion

Previous studies show that the language barrier is significant for immigrants in accessing health and social care services [1,2,3,5,43]. Our study demonstrates that factors such as financial weakness (A25), socio-cultural weakness (A18, A2, A8), and gender weakness (A14 and A30) affect immigrant women’s healthcare accessibility. Especially for childless and aged women, living in Taiwan for over eight years and facing financial pressures were the major obstacles faced in satisfying their healthcare needs. Although most immigrant women in Taiwan feel well, they still have financial anxiety when confronting their healthcare needs.

In the theme “healthcare needs,” the questionnaire items focused on reproductive fertility, including “provide child health checkups,” “provide parental knowledge and pre- and post-natal guidance,” “provide reproductive health knowledge,” “provide information about pre-natal medical allowances,” and “provide medical allowances.” Consequently, “provide medical allowances” (52.53%) was the highest item. This result inspired us to explore which socioeconomic characteristics of the immigrant women were related to “provide medical allowances,” as part of their “healthcare needs.”

**Figure 3:**
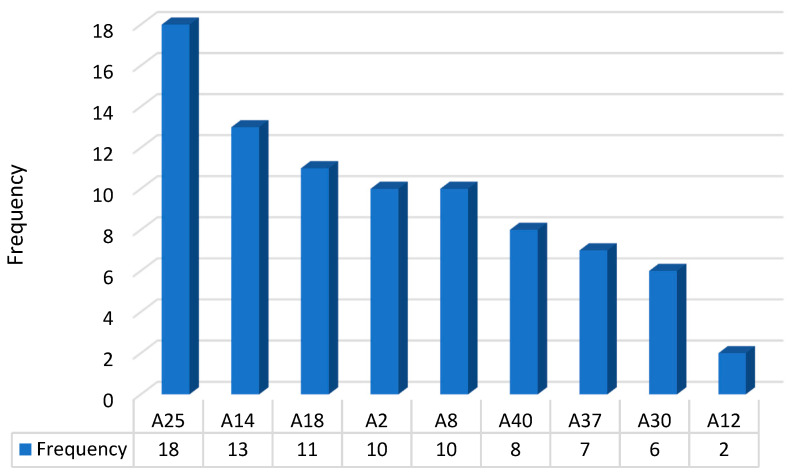
Item frequency in the 26 association rules for healthcare needs (A45: provide medical allowances).

Figure 3 shows the item frequency in the 26 association rules for healthcare needs (A45: provide medical allowance). The most frequent item was A25 (“husband has no job”), followed by the following items: A14 (“marriage frequency is more than twice”), A18 (“educational level of husband is illiterate, self-learned, or primary school”), A2 (“nationality is mainland China”), A8 (“type of identity document is mainland China”), A40 (“agreement of living in Taiwan is safe; rare disasters and accidents”), A30 (“childless with Taiwanese husband”), and A12 (“duration of residence in Taiwan is over 8 years”). These nine items were then classified into four groups for further interpretation (see Table 3): financial weakness (A25), socio-cultural weakness (A18, A2, and A8), gender weakness (A14 and A30), and the feeling of well-being (A40, A37, and A12). Moreover, in Figure 3, financial, socio-cultural, and gender weakness were the main factors leading to the need for medical allowances.

Table 5 presents the top 10 high-confidence association rules by support for healthcare needs (A45: provide medical allowance). According to the table, the following association rules had the highest support: A25 (“husband has no job”), A14 (“marriage frequency is more than twice”), and A2 (“nationality is mainland China”). Meanwhile, Rules 6 and 7 indicated that A30 (“childless with Taiwanese spouse”) was a meaningful characteristic. For childless and aged immigrant women from mainland China, language barriers were less significant than the others, but “providing medical allowances” was the most integral to satisfying their healthcare needs.

In general, graph-based techniques can help visualize association rules using vertices and edges, wherein the former typically represent the items, while the latter indicate the relationship between the rules. Interest measures are also typically added to the plot as labels on the edges or by the color/size of the vertices. However, although graph-based visualizations offer clear representation of the rules, they tend to easily become cluttered and are only viable for small sets of rules [44].

**Figure 4:**
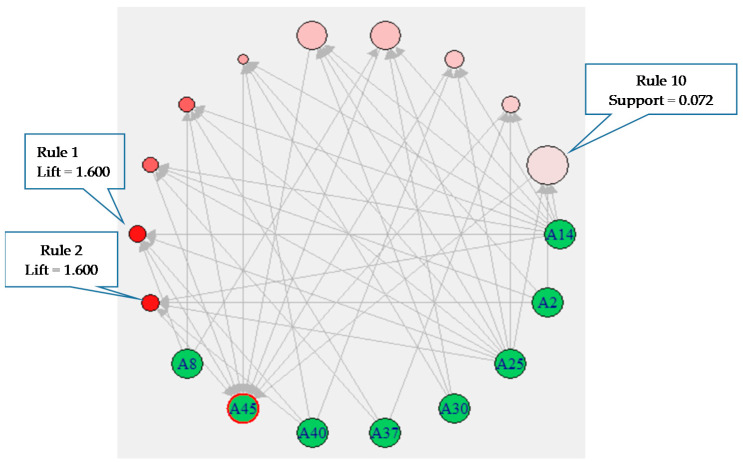
Graph-based visualization, with items for the top 10 high-confidence rules. (Size: support (0.05–0.072); Color: lift (1.582–1.600)).

As shown in Figure 4, we selected the top 10 high-confidence rules and used graph-based techniques to visualize them. In this figure, the largest pink circle represents Rule 10 with the highest support (0.072) and connections to four items: A2, A14, A25, and A45. Meanwhile, the two dark red circles represent Rules 1 and 2 with the highest lift (1.600) and connections to A2, A14, A25, A40, and A45 for Rule 1 and connections to A8, A14, A25, A40, and A45 for Rule 2.

It is important to note that, since 2005, the Taiwanese government has provided health insurance subsidies for economically disadvantaged immigrant women. Despite these subsidies, the majority of the women still answered “provide medical allowances” to the questionnaire item “healthcare needs.” As shown in Figure 4, the main attributes of the population of “provide medical allowances” included A25 (“husband has no job”), A14 (“marriage frequency is more than twice”), A18 (“educational level of husband was illiterate, self-learned, or primary school”), A2 (nationality is mainland China), and A8 (type of identity documents is “mainland China spouses”). According to Table 5, the association rules of the immigrant women’s healthcare needs comprised financial weakness (A25), socio-cultural weakness (A18, A2, and A8), gender weakness (A14 and A30), and the feeling of well-being (A40, A37, and A12). Overall, these results could be interpreted as that, among the immigrant women in this study, being childless and aged, living in Taiwan for over eight years, and facing financial pressure were the major obstacles faced in satisfying their healthcare requirements.

Finally, as reported by some researchers, this study indicated that, upon arrival in the host country, immigrants tend to have lower income levels than the native-born residents [45,46]. Meanwhile, immigrant women represent a diverse group that frequently faces multiple cultural, linguistic, and systemic barriers to adopting and maintaining healthy behaviors. Our findings also confirm previous studies that indicate a strong relevance of the number of years lived in Taiwan to financial pressure, especially for immigrant women from mainland China. Thus, this study demonstrates that the majority of immigrant women’s healthcare needs are due to economic crises.

## 5. Conclusions

This study investigated the relationship between socioeconomic characteristics and healthcare needs among immigrant women in Taiwan. Previous studies indicate that language is a significant barrier for immigrants in accessing health and social care services [1,2,3,5,43]. Our study demonstrates the diverse implications. As shown in Figure 3, financial weakness, socio-cultural weakness, and gender weakness are the main factors resulting in the need for medical allowances. Although most immigrant women in Taiwan feel well, they still have financial anxiety when confronting their healthcare needs. Based on the findings, the barriers faced by immigrant women were related to the limited access to socioeconomic resources and healthcare and poor living conditions. Additionally, the polarization and concentration of wealth and poverty in the social strata were key problems for immigrant families in Taiwan. Meanwhile, the financial anxiety of aged immigrant women was more apparent.

In general, medical care policies for women often over-emphasize the prevention of fertility-related diseases, especially for married immigrant women. As shown in the original survey questionnaire, the theme “healthcare needs” did not include questions on the physical and mental needs of aged women but focused on fertility and child nurture. However, after long-term unequal social and economic living conditions, the financial weakness of aged women has worsened beyond that of men. In fact, an increasing number of vulnerable, aged immigrant women are being forced to live in poor and unhealthy conditions and suffer severe social isolation and resource deprivation. All these issues are associated with various aspects of women’s lives with regard to social, cultural, and economic inequality. Thus, this demographic must endure multiple serious pressures from immigrant adaptation, family roles (e.g., mother and caregiver), and economic disadvantages.

Finally, “economic inequality” and “aging of women’s life cycle” were interrelated issues with immigrant women’s healthcare policies. As shown, women’s immigration and health experiences were quite different from men’s. Therefore, future research should examine such assumptions and reflect on the diverse health experiences of immigrant women. Moreover, policies aimed at protecting the unemployed from poverty, increasing employability, and restoring employment opportunities are necessary steps to prevent the deterioration of immigrant women’s health, especially those who are vulnerable to the effects of economic crises.

## Figures and Tables

**Table 1:** Statistics of healthcare needs (the first choice) among the immigrant women.

Items of Healthcare Needs	*n* = 5848	%
Provide medical allowances	3072	52.53
Provide child health checkups	979	16.74
Provide parental knowledge, and pre- and post-natal guidance	486	8.31
Other needs	1311	22.42

Note. “Other needs” included “provide knowledge of infectious diseases,” “assist medical communication,” “help joining the national health insurance program,” “provide reproductive health knowledge,” and “provide information about pre-natal medical allowances”.

**Table 2:** Descriptive statistics.

Characteristics	*n* = 5848	%	P(Χ^2^)
Nationality			0.000 ***
Southeast Asia, Hong Kong, Macao, other countries	2238	38.27	
Mainland China	3610	61.73	
Educational level			0.000 ***
Illiterate, self-learned, or primary school	1272	21.75	
Junior high school	2256	38.58	
Senior high school	1696	29.00	
College, university, graduate	605	10.35	
Missing value	19	0.32	
Type of identity documents			0.000 ***
Foreign spouses	2186	37.38	
Mainland China spouses	3662	62.62	
Number of years lived in Taiwan			0.000 ***
<4 years	1334	22.81	
4–6 years	1064	18.19	
6–8 years	1291	22.08	
>8 years	2154	36.83	
Missing value	5	0.09	
Marriage frequency			0.000 ***
Once	5040	86.18	
More than twice	783	13.39	
Missing value	25	0.43	
The main source of spending money			0.000 ***
No	150	2.56	
Working income and savings	1921	32.85	
Provided by husband, parents of husband, or children	3709	63.42	
Missing value	68	1.17	
Educational level of husband			0.000 ***
Illiterate, self-learned, or primary school	793	13.56	
Junior high school	1622	27.74	
Senior high school	2378	40.66	
College, university, graduate	990	16.93	
Missing value	65	1.11	
Marriage frequency of husband			0.000 ***
Once	4641	79.36	
More than twice	1184	20.25	
Missing value	23	0.39	
Does husband have a job?			0.000 ***
Yes	4526	77.39	
No	1314	22.47	
Missing value	8	0.14	
Average income of husband			0.000 ***
0~ NT$ 19,999 (US$ 666)	645	11.03	
NT$ 20,000~NT$ 29,999 (US$ 667~US$ 999)	1476	25.24	
NT$ 30,000~NT$ 39,999 (US$ 1000~US$ 1332)	1313	22.45	
Over NT$ 40,000 (US$ 1333)	1029	17.60	
Missing value	1385	23.68	
The number of births with Taiwanese husband			0.000 ***
None	1449	24.78	
One child	2022	35.48	
Two children	2024	34.61	
More children	0	0.00	
Missing value	353	5.13	
The main source of family living expenses			0.835
Self or husband	5541	94.75	
Provided by relatives, children, NGO, government allowances, debit, and credit	248	4.24	
Missing value	59	1.01	
People around me are very friendly			0.070
Disagree	359	6.14	
Agree	4103	70.16	
Highly agree	1382	23.63	
Missing value	4	0.07	
I live in a safe place; disasters and accidents are rare			0.02 *
Disagree	410	7.01	
Agree	4237	72.45	
Highly agree	1198	20.49	
Missing value	3	0.05	
Overall, I am very happy in Taiwan			0.000 ***
Disagree	826	14.12	
Agree	3946	67.48	
Highly agree	1073	18.35	
Missing value	3	0.05	

* *p* < 0.05; *** *p* < 0.001.

**Table 3:** Items regarding the socioeconomic characteristics (X) and healthcare needs (Y) of immigrant women.

Item	Item No.	Descriptions
X (antecedent or left-hand-side, LHS)		Nationality
	A1	Southeast Asia, Hong Kong, Macao, and other countries
	A2	Mainland China
		Educational level
	A3	Illiterate, self-learned, or primary school
	A4	Junior high school
	A5	Senior high school
	A6	College, university, graduate
		Type of identity documents
	A7	Foreign spouses
	A8	Mainland China spouses
		Number of years lived in Taiwan
	A9	<4 years
	A10	4–6 years
	A11	6–8 years
	A12	>8 years
		Marriage frequency
	A13	Once
	A14	More than twice
		The main source of spending money
	A15	No
	A16	Working income and savings
	A17	Provided by husband, parents of husband, or children
		Educational level of husband
	A18	Illiterate, self-learned, or primary school
	A19	Junior high school
	A20	Senior high school
	A21	College, university, graduate
		Marriage frequency of husband
	A22	Once
	A23	More than twice
		Does husband have a job?
	A24	Yes
	A25	No
		Average income of husband
	A26	0~ NT$ 19,999 (US$ 666)
	A27	NT$ 20,000~NT$ 29,999 (US$ 667~US $999)
	A28	NT$ 30,000~NT$ 39,999(US$ 1000~US$ 1332)
	A29	Over NT$ 40,000 (US$ 1333)
		The number of births with Taiwanese husband
	A30	None
	A31	One child
	A32	Two children
	A33	More children
		The main source of family living expenses
	A34	Self or husband
	A35	Provided by relatives, children, NGO, government Allowances; debit and credit
		People around me are very friendly
	A36	Disagree
	A37	Agree
	A38	Highly agree
		I live in a safe place; disasters and accidents are rare
	A39	Disagree
	A40	Agree
	A41	Highly agree
		Overall, I am very happy in Taiwan
	A42	Disagree
	A43	Agree
	A44	Highly agree
Y (consequent or right-hand-side, RHS)		Healthcare needs
	A45	Provide medical allowances
	
	

**Table 4:** The 26 association rule sets by sorting confidence for healthcare needs (A45: provide medical allowances).

Rank/Rule No.	Rule Pattern			Support	Confidence	Lift
1	【A2, A14, A25, A40】	=>	【A45】	0.055	0.841	1.600
2	【A8, A14, A25, A40】	=>	【A45】	0.055	0.841	1.600
3	【A14, A2, A25, A37】	=>	【A45】	0.054	0.838	1.595
4	【A8, A14, A25, A37】	=>	【A45】	0.054	0.838	1.595
5	【A14, A25, A30, A40】	=>	【A45】	0.050	0.835	1.590
6	【A2, A14, A25, A30】	=>	【A45】	0.064	0.833	1.586
7	【A8, A14, A25, A30】	=>	【A45】	0.064	0.833	1.586
8	【A14, A25, A40】	=>	【A45】	0.056	0.833	1.585
9	【A14, A25, A37】	=>	【A45】	0.055	0.832	1.584
10	【A2, A14, A25】	=>	【A45】	0.072	0.831	1.582
11	【A8, A14, A25】	=>	【A45】	0.072	0.831	1.582
12	【A14, A25, A30】	=>	【A45】	0.065	0.827	1.574
13	【A8, A18, A25】	=>	【A45】	0.060	0.825	1.570
14	【A14, A25】	=>	【A45】	0.073	0.824	1.569
15	【A2, A18, A25】	=>	【A45】	0.059	0.824	1.569
16	【A8, A18, A37, A40】	=>	【A45】	0.051	0.818	1.557
17	【A2, A18, A37, A40】	=>	【A45】	0.051	0.817	1.556
18	【A2, A12, A25】	=>	【A45】	0.067	0.817	1.555
19	【A8, A12, A25】	=>	【A45】	0.067	0.815	1.551
20	【A18, A25】	=>	【A45】	0.066	0.810	1.542
21	【A8, A18, A30】	=>	【A45】	0.053	0.809	1.541
22	【A2, A18, A30】	=>	【A45】	0.053	0.809	1.540
23	【A8, A18, A37】	=>	【A45】	0.059	0.803	1.529
24	【A2, A18, A37】	=>	【A45】	0.059	0.803	1.528
25	【A8, A18, A40】	=>	【A45】	0.061	0.802	1.527
26	【A2, A18, A40】	=>	【A45】	0.061	0.802	1.526

**Table 5:** Top 10 high-confidence association rules by support for healthcare needs (A45: provide medical allowance).

Rule No.	Item	Rule Pattern			Support	Confidence	Lift
10	4	【A2, A14, A25】	=>	【A45】	0.072	0.831	1.582
6	5	【A2, A14, A25, A30】	=>	【A45】	0.064	0.833	1.586
7	5	【A8, A14, A25, A30】	=>	【A45】	0.064	0.833	1.586
8	4	【A14, A25, A40】	=>	【A45】	0.056	0.833	1.585
9	4	【A14, A25, A37】	=>	【A45】	0.055	0.832	1.584
1	5	【A2, A14, A25, A40】	=>	【A45】	0.055	0.841	1.600
2	5	【A8, A14, A25, A40】	=>	【A45】	0.055	0.841	1.600
3	5	【A14, A2, A25, A37】	=>	【A45】	0.054	0.838	1.595
4	5	【A8, A14, A25, A37】	=>	【A45】	0.054	0.838	1.595
5	5	【A14, A25, A30, A40】	=>	【A45】	0.050	0.835	1.590

## Data Availability

The secondary data in this study were obtained from the “Survey of Foreign and Chinese Spouses’ Living Requirements, 2008,” which was administered by the Ministry of the Interior, Taiwan. The data distribution was performed by Academia Sinica, a survey research data archive.
